# Tuberculosis of the Iliac Bone and Acetabulum With Iliopsoas, Obturator Internus, and Obturator Externus Abscesses in an Immunocompetent Indian Female: An Extremely Rare Case

**DOI:** 10.7759/cureus.55727

**Published:** 2024-03-07

**Authors:** Sankalp Yadav

**Affiliations:** 1 Medicine, Shri Madan Lal Khurana Chest Clinic, New Delhi, IND

**Keywords:** fine needle aspiration cytology (fnac), cbnaat/ xpert/ rif assay, immunocompetent, externus abscesses, obturator internus, ilio-psoas, acetabulum, iliac bone, mtb, tuberculosis

## Abstract

Skeletal tuberculosis is a rare form of extrapulmonary tuberculosis. Due to non-specific clinical features, these cases are often diagnosed very late, ultimately affecting treatment outcomes. The present case is a very rare case of tuberculosis of the iliac bone and acetabulum with iliopsoas, obturator internus, and obturator externus abscesses in an Indian female. She reported pain in her right hip and a limp. It was a difficult diagnosis, especially due to the rare involvement of bones and muscles in the absence of any lesions in the lungs. Nevertheless, the diagnosis was achieved by a detailed radiometric and laboratory workup. She was initiated on antituberculous treatment per her weight for 12 months.

## Introduction

Tuberculosis is one of the oldest known diseases and is still one of the leading causes of mortality and morbidity [1]. Extrapulmonary tuberculosis is an outcome of infection caused by *Mycobacterium tuberculosis* in locations other than the lungs [2]. This type of tuberculosis constitutes 10-15% of the total tuberculosis cases [3]. Besides, nearly 3% of cases of tuberculosis as a whole and 10-30% of cases of extrapulmonary tuberculosis have been linked to the musculoskeletal system [4].

In extrapulmonary tuberculosis, the most common sites are the lymph nodes, followed by the skeletal system [1]. Of all the tuberculosis cases, the incidence of skeletal tuberculosis is less than 5% [5]. The present case is an extremely rare case of simultaneous involvement of the iliac bone and acetabulum with iliopsoas, obturator internus, and externus abscesses in a 15-year-old Indian female.

## Case presentation

A 15-year-old non-diabetic Indian female reported to the outpatient department in 2022 with complaints of pain in the right hip and difficulty walking. She was alright six months ago when she fell down the stairs. She consulted a local clinician at that time, who advised painkillers, and her pain subsided. However, during the subsequent months, her pain aggravated on walking and relieved (slightly) when she rested. It was associated with a limp. There were no discharging sinuses or swelling.

There were no other constitutional symptoms of tuberculosis, a history of disease in the family, or any contacts. She was a student belonging to a low socioeconomic background. However, there was no history of substance abuse or stays at night shelters or refugee camps.

General examination revealed an afebrile female with a pulse of 68/minute, blood pressure of 120/70 mmHg, respiratory rate of 16/minute, and peripheral capillary oxygen saturation (SpO_2_) of 99% on room air. Her systemic examination was unremarkable. Local examination was suggestive of tenderness in the right anterior and posterior hip joints. There were restricted joint movements with flexion and external and internal rotations that were terminally restricted and painful. Further, there was no clubbing, icterus, pallor, lymphadenopathy, cyanosis, edema, or koilonychia.

A detailed lab workup revealed a complete leucocyte count of 5900/mm^3^, consisting of 70% polymorphonuclear cells and 29% lymphocytes. Hemoglobin was 10.5 g/dl. C-reactive proteins and erythrocyte sedimentation rate (first hour) were 31 mg/l and 67 mm, respectively. *Mycobacterium* culture and acid-fast bacilli direct smear of induced sputum yielded negative results. Additionally, she tested negative for HIV (I and II).

**Figure 1:**
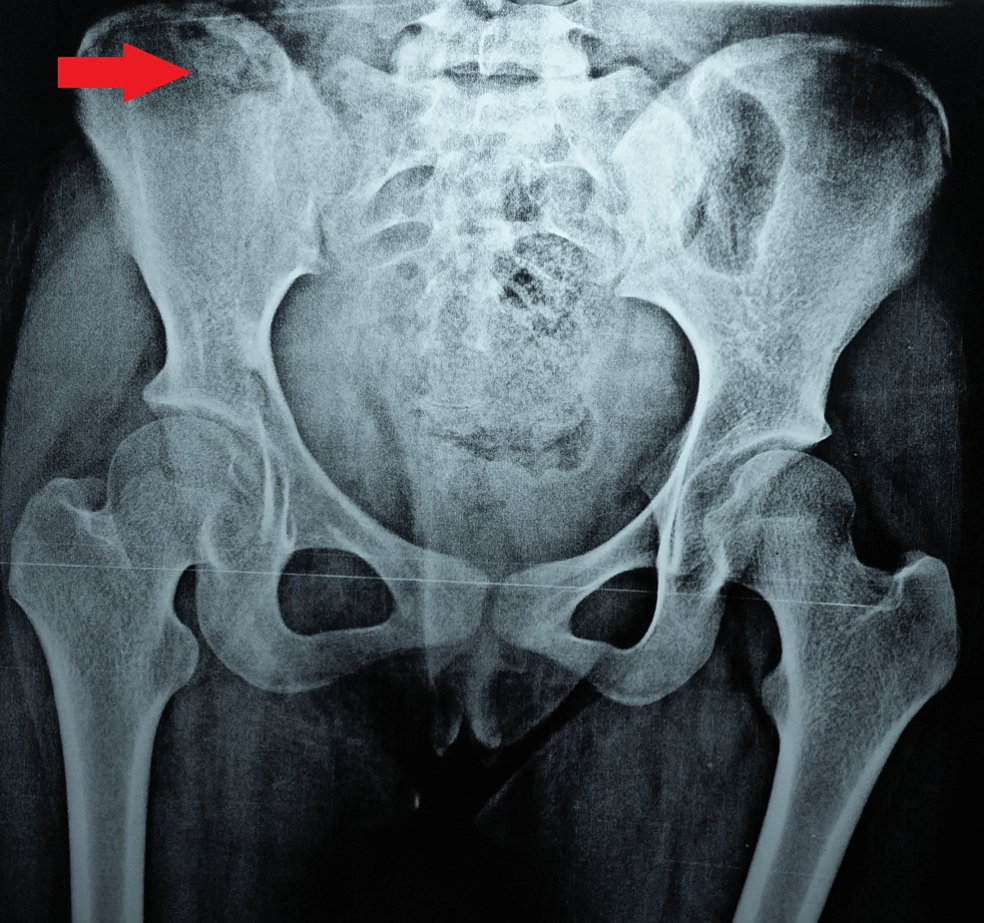
Plain radiograph of the pelvis (AP view) AP: anteroposterior

A plain radiograph of the pelvis showed a well-defined diffuse lytic lesion in the body of the right iliac bone (Figure 1).

**Figure 2:**
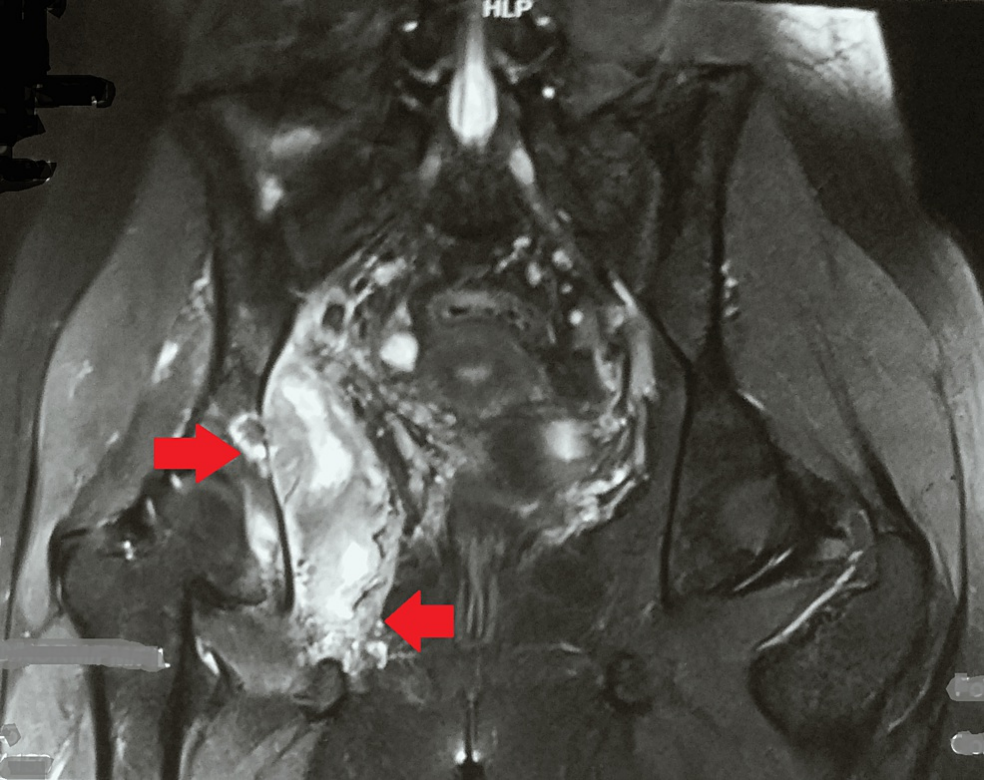
MRI of the right hip joint showing a large multiloculated intercommunicating collection/abscess in the right iliopsoas muscles, obturator internus, and obturator externus MRI: magnetic resonance imaging

**Figure 3:**
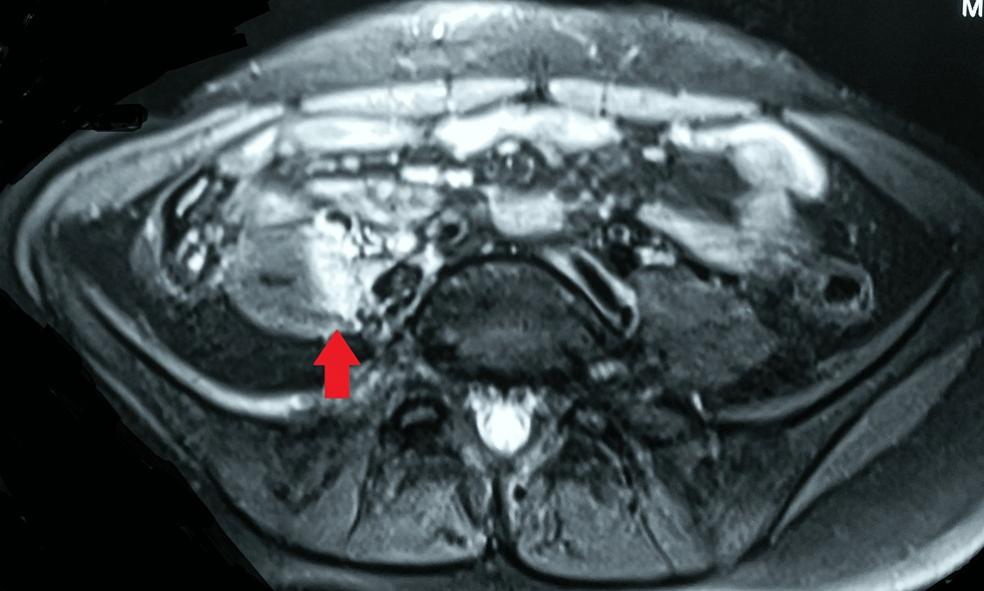
MRI showing the abscess MRI: magnetic resonance imaging

**Figure 4:**
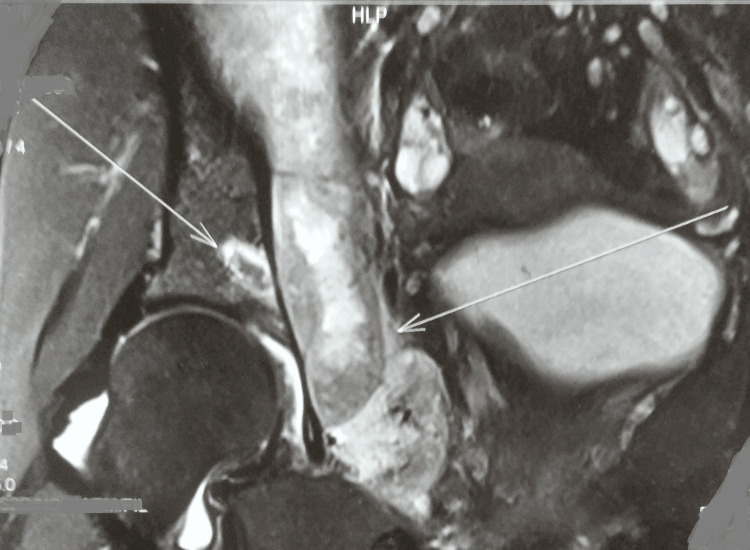
MRI of the right hip showing the lesions and abscesses MRI: magnetic resonance imaging

A chest radiograph was normal. Magnetic resonance imaging of the right hip joint indicated a large multiloculated intercommunicating collection/abscess (15.5 x 8.5 x 9 cm) involving the right iliopsoas muscles, obturator internus, and obturator externus, with few internal septae and surrounding soft tissue edema. Moreover, there was mild hip joint effusion with marrow edema along with erosions involving the anterior and posterior columns of the right acetabulum and iliac bone (Figures 2-4).

**Figure 5:**
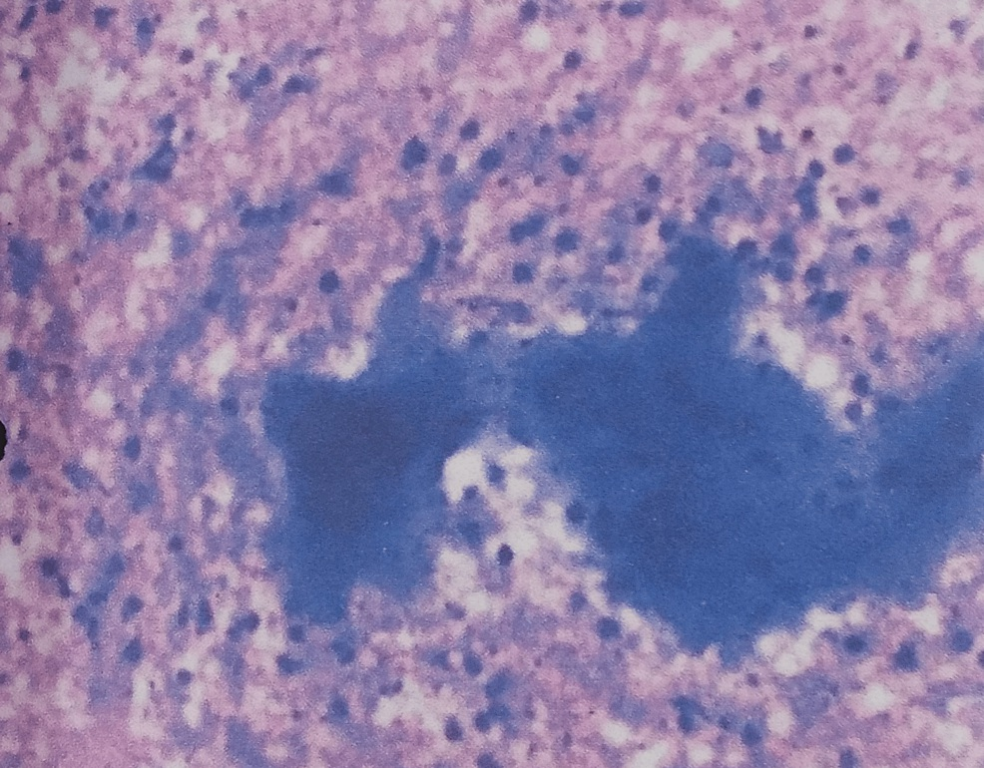
USG-guided FNAC of the pus showing necrosis USG: ultrasound; FNAC: fine-needle aspiration cytology

An ultrasound-guided fine needle aspiration cytology of the pus was done from the iliopsoas region, and about 30 ml of thick purulent fluid was drained. Smears of the pus revealed an acute suppurative lesion with predominantly necrosis along with viable and degenerate polymorphs and phagocytic histiocytes. No granuloma was seen, and stains for acid-fast bacillus were negative (Figure 5).

**Figure 6:**
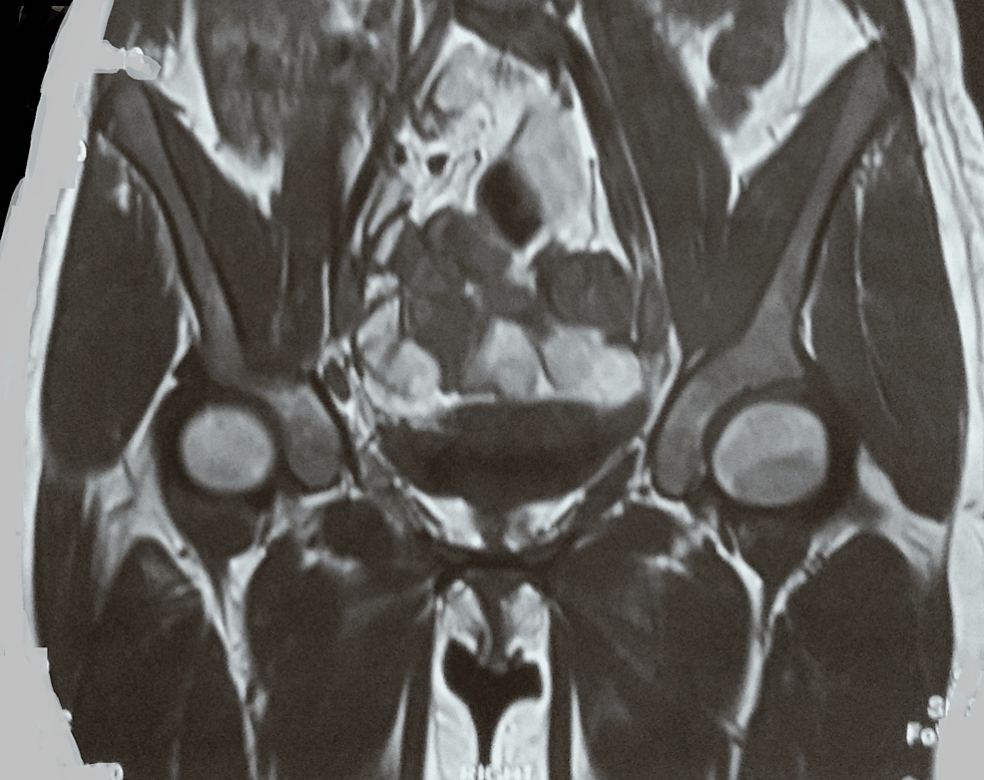
MRI of the right hip showing complete resolution of the disease MRI: magnetic resonance imaging

The cartridge-based nucleic acid amplification test of the pus was suggestive of low detection of *Mycobacterium tuberculosis* with no resistance to rifampicin. A diagnosis of tuberculosis of the iliac bone and acetabulum with iliopsoas, obturator internus, and obturator externus abscesses was made, and an antituberculous treatment per her weight was initiated with fixed-dose combinations of rifampicin, pyrazinamide, ethambutol, and isoniazid. Additionally, she was regularly counseled for treatment adherence and followed up on a timely basis in both the orthopedics and infectious diseases outpatient departments. After the completion of 168 days of antituberculous treatment, her treatment was extended to a total of 12 months per the national guidelines and the orthopedician's opinion. She completed her treatment successfully without any adverse drug reactions, and a repeat magnetic resonance imaging of the right hip joint after 12 months of antituberculous treatment was suggestive of no significant abnormality with complete resolution of the disease process. Although there was improvement in her condition with a resolution of pain, a slight limp was present on walking, for which she was advised to seek physiotherapy (Figure 6).

## Discussion

Musculoskeletal tuberculosis is a relatively rare entity (1-3% of total tuberculosis cases) [6]. Almost half of the cases of skeletal tuberculosis involve the spine. Other sites are the hip joint, knee, wrist, elbow, sacroiliac joint, sacrum, and pubic bones [7].

Hematogenous transmission from the primary infected focus is typical for tuberculous osteomyelitis. Both in the lungs and other viscera, the main focus can be either active or quiescent, latent or apparent [7]. However, lymphatic spread or direct inoculation due to trauma can also result in the disease. Additionally, only around 50% of cases of skeletal tuberculosis have foci in the lungs [7,8]. Therefore, in patients with normal chest radiographs, as seen in the present case, there are chances of a delayed diagnosis, which may be due to a lack of awareness among the primary care clinicians.

The involvement of bones like the iliac bone and the acetabulum itself is a very rare presentation. Further, with less than 1% of cases of skeletal tuberculosis, primary tuberculous abscess in muscles is uncommon [9]. Pyomyositis is a very rare condition, as striated muscles have a low oxygen content, a high lactic acid content, and an absence of reticuloendothelial system components, which makes them resistant to mycobacterial infection [10]. It is commonly caused by *Staphylococcus aureus* (70-90%). Other causative organisms include *Streptococcus pyogenes*, *Neisseria gonorrhoeae*, *Enterococcus faecalis*, *Escherichia coli*, *Salmonella enteritidis*, and *Mycobacterium tuberculosis* [11]. Purulent discharges in the iliopsoas muscle compartment are indicative of a psoas abscess [12]. It has a yearly incidence of roughly 12 cases worldwide. Most of these cases are brought on by infections with Staphylococcus aureus. An iliopsoas abscess may sporadically result from *Mycobacterium tuberculosis*. The sluggish start and occult characteristics of tuberculosis, along with its generic clinical presentation, can lead to a misdiagnosis of psoas abscess [4]. Further, pyomyositis of the obturator externus and obturator internus is an exceedingly rare presentation and can afflict any age group, including those in good health [11]. This condition mimics other more prevalent conditions in this location, such as transient synovitis of the hip and septic arthritis, resulting in a diagnostic dilemma [11].

Diagnosis is mainly based on advanced radiometric techniques like magnetic resonance imaging, fine-needle aspiration cytology, cartridge-based nucleic acid amplification tests, and the culture of the pus to isolate the bacteria. Further ruling out drug resistance is essential for the proper management of such cases. Management is essentially medical with antituberculous drugs. However, in cases with large pus collections or impending bony destruction, percutaneous drainage with corrective surgeries is indicated [13].

A case of tuberculosis of the iliac bone and acetabulum with iliopsoas, obturator internus, and obturator externus abscesses in an immunocompetent is never reported in the medical literature. This case stresses the importance of reporting and documentation of similar cases; this will not only help in the timely management of such cases but will also avert any untoward consequences.

## Conclusions

An extremely rare case of tuberculosis of the iliac bone and acetabulum with iliopsoas, obturator internus, and obturator externus abscesses in an immunocompetent female is reported here. This case sheds light on the importance of an eye for rare presentations like this among primary care clinicians. The diagnostic delays due to a lack of awareness even in endemic countries could result in permanent disfigurement or the development of drug-resistant tuberculosis.

## References

[1] Yadav S (2022). Iliac bone tuberculosis presenting as left thigh swelling in an Indian female patient: a rare case. Cureus.

[2] Lee JY (2015). Diagnosis and treatment of extrapulmonary tuberculosis. Tuberc Respir Dis (Seoul).

[3] Jain M, Sarkar S, Naik S, Behera S (2018). Iliac bone tuberculosis with bicompartmental abscess. BMJ Case Rep.

[4] Mohandes AF, Karam B, Alrstom A (2022). Primary psoas tuberculosis abscess with an iliac bone lytic lesion: a case report. J Med Case Reports.

[5] Elghoul N, Benchakroun M, Zaddoug O, Bennis A, Zine A, Tanane M, Jaafar A (2020). A report of two challenging cases of bone infection: Mycobacterium tuberculosis. How to manage?. Oxf Med Case Reports.

[6] Saraf SK, Tuli SM (2015). Tuberculosis of hip: a current concept review. Indian J Orthop.

[7] Ismail M, Szmigielski W, Sinha NR (2009). Isolated iliac bone tuberculosis: a case report. Pol J Radiol.

[8] Davidson PT, Horowitz I (1970). Skeletal tuberculosis: a review with patient presentations and discussion. Am J Med.

[9] Held MF, Hoppe S, Laubscher M, Mears S, Dix-Peek S, Zar HJ, Dunn RN (2017). Epidemiology of musculoskeletal tuberculosis in an area with high disease prevalence. Asian Spine J.

[10] Batra S, Ab Naell M, Barwick C, Kanvinde R (2007). Tuberculous pyomyositis of the thigh masquerading as malignancy with concomitant tuberculous flexor tenosynovitis and dactylitis of the hand. Singapore Med J.

[11] Khoshhal K, Abdelmotaal HM, Alarabi R (2013). Primary obturator internus and obturator externus pyomyositis. Am J Case Rep.

[12] Mallick IH (2004). Iliopsoas abscesses. Postgrad Med J.

[13] Vasigh M, Karoobi M, Montazeri M, Moradi G, Asefi H, Gilani A, Meshkati Yazd SM (2022). Isolated psoas abscess caused by mycobacterium tuberculosis: a rare case report. Clin Case Rep.

